# A multimodal MRI dataset of professional chess players

**DOI:** 10.1038/sdata.2015.44

**Published:** 2015-09-01

**Authors:** Kaiming Li, Jing Jiang, Lihua Qiu, Xun Yang, Xiaoqi Huang, Su Lui, Qiyong Gong

**Affiliations:** 1 Huaxi MR Research Center (HMRRC), Departments of Radiology, West China Hospital of Sichuan University, Chengdu, Sichuan 610041, China; 2 School of Sociality and Psychology, Southwest University for Nationalities, Chengdu, Sichuan 610041, China; 3 Department of Psychology, School of Public Administration, Sichuan University, Chengdu, Sichuan 610041, China

**Keywords:** Problem solving, Brain imaging, Magnetic resonance imaging, Functional magnetic resonance imaging

## Abstract

Chess is a good model to study high-level human brain functions such as spatial cognition, memory, planning, learning and problem solving. Recent studies have demonstrated that non-invasive MRI techniques are valuable for researchers to investigate the underlying neural mechanism of playing chess. For professional chess players (e.g., chess grand masters and masters or GM/Ms), what are the structural and functional alterations due to long-term professional practice, and how these alterations relate to behavior, are largely veiled. Here, we report a multimodal MRI dataset from 29 professional Chinese chess players (most of whom are GM/Ms), and 29 age matched novices. We hope that this dataset will provide researchers with new materials to further explore high-level human brain functions.

## Background and Summary

Chess is a very demanding board game^1,2^. It taxes several mental resources of players, including spatial cognition, memory, planning, and problem solving^3^. As a good model to investigate high-level human brain functions, it has been studied for decades^4–7^. Recently, advances in brain imaging techniques, e.g., positron emission tomography (PET)^8^, diffusion tensor imaging (DTI)^9^, and functional magnetic resonance imaging (fMRI)^10^, have greatly facilitated this area of research by providing in-vivo brain images of chess players. For instance, Atherton *et al.*, conducted a task-based fMRI experiment on amateur chess players and found that during the game, cortical regions for attention, spatial perception, imagery and mental rotation were active^2^. Wan *et al.*, also employed fMRI techniques to explore neural correlates of next-move generation, and found that the best next-move generation activated the *precuneus-caudate* circuit in board game experts^11^. These studies demonstrate that non-invasive MRI techniques are valuable tools to investigate the underlying neural mechanism of playing chess.

Another particularly interesting question regarding chess, is how long-term practice/training sculpts the brains of professional players. According to Gobet’s survey, it takes on average seven years of serious practice to become a master in chess^1^. Since the human brain is able to adapt in response to learning^12–14^, it is reasonable to expect professional chess GM/Ms to exhibit fundamental neurological changes. This has been demonstrated by a voxel-based morphometry (VBM)^15^ study, in which Duan *et al.*, found that the bilateral caudate nuclei of GM/Ms are significantly smaller than those of normal people^16^. Beside the structural alteration, the default mode network (DMN)^17^ of GM/Ms demonstrated broader deactivation than that of novices^18^, and the functional connections between basal ganglia, thalamus, hippocampus, and several parietal and temporal regions were reported to be increased in GM/Ms compared with novices^19^. However, what are the structural and functional alterations of GM/Ms due to long-term professional practice, and how these alterations relate to their behavior are still largely veiled, and therefore need systematic investigation.

Therefore, we report here on a multimodal MRI dataset of 29 professional Chinese chess (Chinese chess is very similar to the international chess. Both are adaptions of Shatranj.) players, mostly GM/Ms, and 29 age matched novices. For each subject, T1 structural MRI, resting state fMRI and DTI data were collected using a Siemens 3T TRIO system. A battery of phenotypic data, including rating and training time, was also collected for the professional players. Additionally this dataset has been used in three prior publications^16,18,19^. We hope that the public availability of this dataset will provide researchers with new material to further explore high-level human brain functions.

## Methods

### Participants

Twenty nine Chinese chess players (age: 28.72±10.84; 9 females) were recruited from the first National Intelligence Games held in 2009 at Chengdu, China. These participants were professional chess players that had been taking serious training regularly (training time: 4.24±1.73 hours/day). In particular, seventeen of them were rated as GM/Ms, and twenty-three scored over 2200 on Elo’s chess-skill rating scale^20^ (Elo scale: 2401.09±134.58), which is an entry level for chess masters by the standards of United States Chess Federation. Meanwhile, twenty nine novices (age: 25.76±6.95; 15 females) with limited chess knowledge/skills were recruited as the control group. The two groups were matched for ages (*P*=0.2016). All participants from both groups are right handed and have no history of mental or physical disorders. Their detailed information can be found at the phenotypic data in Supplementary File 1 and 3.

The Research Ethics Committee of West China Hospital of Sichuan University approved this study. Informed consents were collected from all participants prior to questionnaires and MRI sessions.

### Experimental design

#### Phenotypic data

For each subject, a series of phenotypic data was collected. These data include regular demographics for typical MRI study including age, sex, education, weight, handedness, and mental and physical illness. Besides, for professional players, some chess-related information was also recorded, including the certified game rate in 2009, the Elo’s chess-skill rating scale, starting age for chess, starting age for professional practice, and how much practice time in a day, a week, a month and a year. In particular, the Raven’s progressive matrices (RPMs) test^21^ was given to each player to evaluate his/her reasoning ability. Specifically, 60 RPMs were displayed on a computer screen using Microsoft PowerPoint. Before these RPMs, two instruction slides on how to perform the test were given to each subject. Following the instructions were two illustrations where A1 and A2 of the RPMs were used as examples. The subject was then asked to finish the remaining test independently, and his/her final score was recorded. For novices, their knowledge/skills about Chinese chess were collected. All above-mentioned information was summarized in the phenotypic data table in Supplementary File 1 and 2.

#### MRI data

For each subject, three sessions of MRI data were collected from a Siemens 3T TRIO system (Siemens, Erlangen, Germany) at West China Hospital of Sichuan University, Chengdu, China. First, a 1 mm isotropic high-resolution T1-weighted structural image or sMRI was acquired using an MPRAGE sequence. The parameters are: TR=1900 ms, TE=2.26 ms, TI=900 ms, Bandwidth=200 Hz/Px, Fov=256*256 mm^2^, Flip angle=9°, 176 slices (please refer to Supplementary File 3 for detailed protocol information).

Then a resting state fMRI or rfMRI scan was performed using an EPI sequence, with TR=2000 ms, TE=30 ms, Bandwidth=2442 Hz/Px, Fov=240*240 mm^2^, Flip angle=90°, slice thickness=5 mm (please refer to Supplementary File 4 for detailed protocol information). Each volume was composed of thirty 64*64 slices that were acquired in an interleaved fashion (i.e., 0,2,4,···,1,3,5, ···). 205 volumes were collected, resulting in a 6 min 50 s rfMRI scan for each subject. During the scan, participants were instructed to look at the fixation cross, minimize motion, and to not think of anything particular.

Lastly, two runs of diffusion tensor images were collected using an EPI sequence. For each run, one b0 image and 20 diffusion-weighted images were acquired. The imaging parameters are as follows: TR=6800 ms, TE=93 ms, GRAPPA acceleration factor=2, Bandwidth=1396 Hz/Px, Fov=230*230 mm^2^, and Voxel size=1.8*1.8*3.0 mm^3^. Detailed protocol information can be found at Supplementary File 5.

## Data Records

This dataset is publicly available at Li *et al.*, 2015 (Data Citation 1). The MRI and QA data can be accessed at http://fcon_1000.projects.nitrc.org/indi/pro/wchsu_li_index.html. The total size of this dataset is approximately 60GB. It contains raw MRI data, QA reports, and QA extras. These data were separately packaged and compressed so that users can download necessary packages in a multi-thread way. The contents and data structures of these packages are detailed as follows.

### Raw MRI data

The raw MRI data for each subject includes an sMRI image (named anonymized.nii.gz), rfMRI images (named rest.nii.gz), and DTI images (named dti.nii.gz). These data were put into three separate folders (anat, rest, and dti respectively), packaged with compression and named raw.subx.chess.tar.gz for a subject x in the chess group or raw.subx.ctrl.tar.gz for a subject x in the control group. Please note that b-values and b-vectors were also put in the dti folder with names as dti.bval and dti.bvec, respectively.

### QA report

The package qaReport.tar.gz is an all-in-one package of QA analysis performed in the present study for all modalities and subjects. All QA results were integrated into webpages, and linked in an overview page (index.html). Specifically, this package includes the overview html webpage, and two folders (named as chess and ctrl for the chess and control group respectively). Each folder has four sub-folders, namely t1QA, restQA1, restQA2, and dtiQA, for modality-specific QA analysis results. The t1QA folder contains t1 QA analysis results using the FreeSurfer QA Tools (QAtools_v1.1.tar, https://surfer.nmr.mgh.harvard.edu/fswiki/QATools), which are mainly screenshots of various volumes/surfaces resulting from the recon-all pipeline. The restQA1 folder is composed of rfMRI QA analysis results using an in-house pipeline, while the restQA2 contains QA results using the BXH/XCEDE Tools (https://www.nitrc.org/projects/bxh_xcede_tools). The dtiQA folder has DTI QA reports in PDF formats, with each PDF file corresponding to one subject. For t1QA and restQA1 that have complex data structures, the results of each subject were put into a separate folder named after his/her ID; while for restQA2 and dtiQA, the results for all subjects were integrated into a single folder due to the simplicity. This package is about 2GB in size, and can be accessed directly at ftp://www.nitrc.org/fcon_1000/htdocs/wchsu_li_chess/qaReport.tar.gz.

### QA extras

This package has results and intermediate files of performed QA analysis. It contains three folders, i.e., t1, rest and dti, corresponding to sMRI, rfMRI and DTI data, respectively. For a general subject x in the chess group, the compressed files are named as qa.extras.t1.subx.chess.tar.gz, qa.extras.rest.subx.chess.tar.gz and qa.extras.dti.subx.chess.tar.gz for t1, rest and dti folders, respectively. The file qa.extras.t1.subx.chess.tar.gz contains files generated by the FreeSurfer recon-all pipeline; the qa.extras.rest.subx.chess.tar.gz contains intermediate files of our in-house rfMRI QA pipeline; and the qa.extras.dti.subx.chess.tar.gz has intermediate files of the DTI QA pipeline (see below for details). For the control group, files are similarly named, with chess replaced by ctrl in the filenames.

### Phenotypic data

Beside the MRI data, the phenotypic information of this dataset was integrated into Supplementary File 1 and 2. It has basic demographics including sex, age, education and weight. For professional chess players, it also includes chess-related data, such as the Elo’s chess-skill rating scale, and professional training time per day, week, month and year.

## Technical Validation

In this section, we performed several QA analyses to evaluate the quality of acquired MRI data. All data were first converted from DICOM format to NIFTI format using the dcm2nii DICOM converter, a part of MRIcron by Chris Rorden (http://www.mccauslandcenter.sc.edu/mricro/mricron/). All subsequent QA analyses were based on NIFTI format with compression (suffix: nii.gz). ***It needs to note that only summarization information will be shown here. Most QA reports described in this section are integrated into the webpage package qaReport.tar.gz, which can be downloaded at*** ftp://www.nitrc.org/fcon_1000/htdocs/wchsu_li_chess/qaReport.tar.gz. It also needs to note that the IDs of subjects reported here are not the same with those in the FCP/ INDI repository, since the latter are too long, making them inappropriate in figures. Nevertheless, there is a simple constant difference, i.e., 28196, between the two ID systems. For the chess group, IDs reported in the descriptor start from 1 to 29, while the IDs in the repository start from 0028197 to 0028225. For the control group, IDs in the descriptor start from 101 to 129, and they start from 0028297 to 0028325 in the repository. Please refer to Supplementary File 6 for all ID mappings.

### 3D structural MRI (sMRI)

The high-resolution sMRI data was first anonymized using NiftiAnonymizer (FullAnonymize.sh V1.0b; http://www.nitrc.org/frs/shownotes.php?release_id=1902). All identifying information in the NIFTI header was removed. Faces, if recognizable, were masked to minimize the risk of re-identification. The anonymized sMRI image was then sent to the FreeSurfer pipeline (https://surfer.nmr.mgh.harvard.edu/fswiki/FreeSurferWiki, version: v5.3.0 stable) for surface reconstruction and registration. This pipeline took about 10 h on our computer (CPU: Intel(R) Xeon(R) E5–4620 v2 @ 2.60 GHz) and all subjects in this dataset went through without errors.

After the pipeline, the FreeSurfer QA Tools were executed to assess the recon quality, to verify file existence and generation order, and to take snapshots of various volumes and surfaces for visual inspection. All subjects in our dataset passed the FreeSurfer QA assessment. The resultant snapshots have been visually inspected for image quality issues and recon errors by experts. Two subjects (sub18 and sub20) seem to have noticeable motions artifacts, although their tissue segmentations and surface reconstructions appear decent.

**Figure 1:**
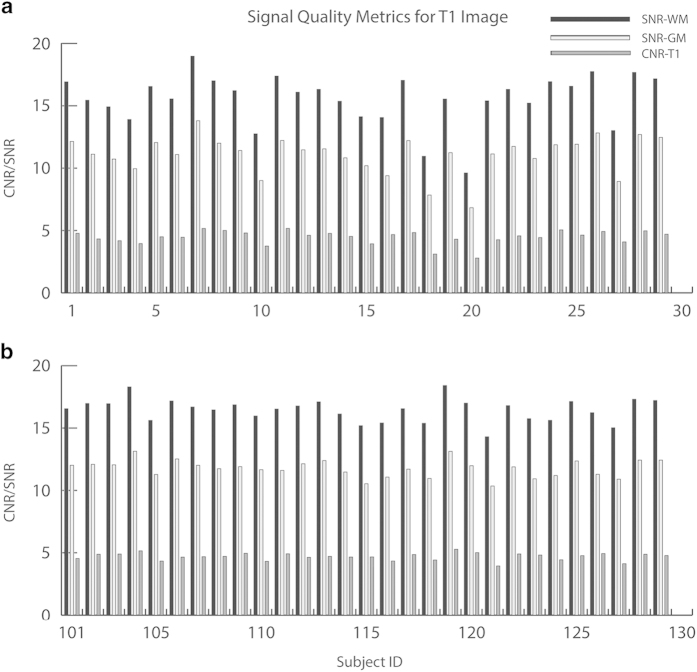
Signal quality metrics examined in present study for T1-weighted MRI images, including SNR for white matter (WM) and gray matter (GM), and Contrast (WM/GM) to Noise Ratio (CNR) for t1 images. Noise level was estimated using non-body background in the T1 image. Panel a: the chess group. Subject ID starts from 1 here or 0028197 in the repository. Panel b: the control group. Subject ID starts from 101 here or 0028297 in the repository.

In addition to the FreeSurfer QA assessment, we also calculated the signal-to-noise ratio or SNR of white matter (WM) and gray matter (GM), and the WM/GM contrast to noise ratio (CNR) using in-house software. The results were summarized in Fig. 1, and were integrated in the overview QA page (index.html, please refer to the data records section for details). As depicted in panel a of Fig. 1, our results show subjects 18 and 20 (repository ID: 0028214 and 0028216) in the chess group have suboptimal GM SNR, WM SNR and WM/GM contrasts, which are consistent with results from the FreeSurfer QA tools. Nevertheless, other subjects in the chess group have decent WM SNRs, GM SNRs and WM/GM CNRs, which are averaged at 15.54±2.05, 11.08±1.53, and 4.46±0.56, respectively. For the control group, all sMRI images have decent quality, as shown in panel b of Fig. 1. The WM SNRs, GM SRNs and WM/GM CNRs are averaged at 16.46±0.93, 11.76±0.69, and 4.70±0.30, respectively.

### Resting-state fMRI

Resting state fMRI data quality was first assessed using the BXH/XCEDE Tools from the fBIRN project (https://www.nitrc.org/projects/bxh_xcede_tools). This package calculates quantitative metrics listed in Table 1, and generates group-wise statistics of these metrics. To perform this analysis, NIFTI images were first converted to AFNI format (BRIK+HEAD), and then xml based header files (.bxh) were generated using the provided afni2bxh converter in the BXH/XCEDE package. After that, the pipeline fmriqa_generate.pl was executed via command: fmriqa_generate.pl --indexjs --verbose --forcetr=2 –defergroup *.bxh outputDir. An html-based report was then generated, and can be found at the restQA2 folder in the QA report package.

Since this tool doesn’t provide all commonly used metrics in fMRI study, e.g., registration performance, we conducted further QA analysis using an in-house shell script, with helps from some third-party software packages, including FSL (http://www.fmrib.ox.ac.uk/fsl, version: v5.0.6), FreeSurfer, AFNI (http://afni.nimh.nih.gov/afni, version: AFNI_2011_12_21_1014), and C-PAC (https://github.com/FCP-INDI/C-PAC, version: v0.3.8). The additionally examined QA items by our script are listed in Table 2.

The preprocessing of rfMRI follows the script (http://www.nitrc.org/frs/downloadlink.php/2628, version: 1.1) released by the 1000 functional connectome project. Specific processing includes: 1) Dropping the first five volumes; 2) Deobliquing the data; 3) Reorienting the images into the RPI coordinate space; 4) Motion correction; 5) Skull removal; 6) Low frequency drift removal using a 2^nd^ order polynomial model; 7) Spatial smoothing using a Gaussian kernel with FWHM=6 mm; 8) Temporal filtering using a band-pass filter with bandwidth 0.01~0.1 Hz; 9) Nuisances (including motion parameters (3 rotation, 3 translation), mean WM signal, and mean CSF signal) removal using a general linear regression model; 10) Registration of functional images to the high-resolution sMRI image and the MNI 152 template.

**Figure 2:**
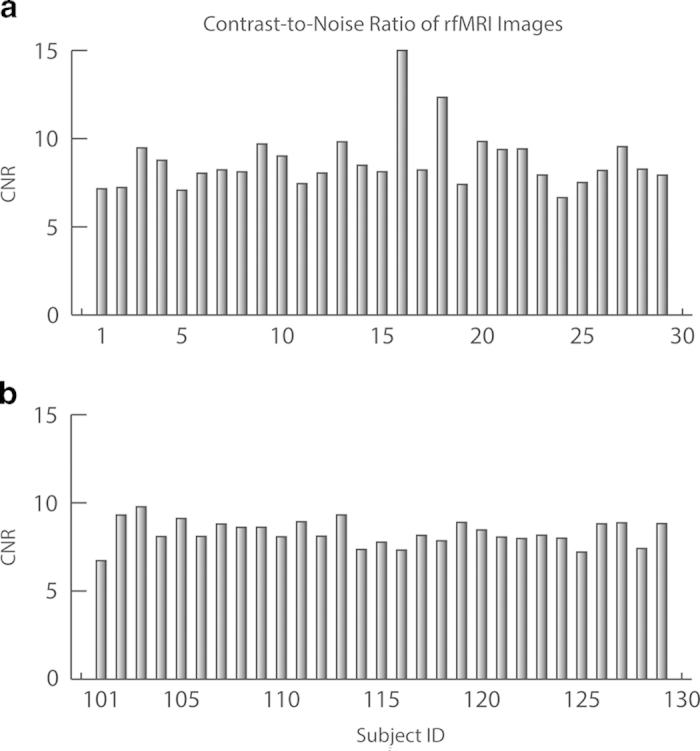
CNRs of the rfMRI images. The noise level was estimated using non-body background in the rfMRI images. (**a**) the chess group. (**b**) the control group.

**Figure 3:**
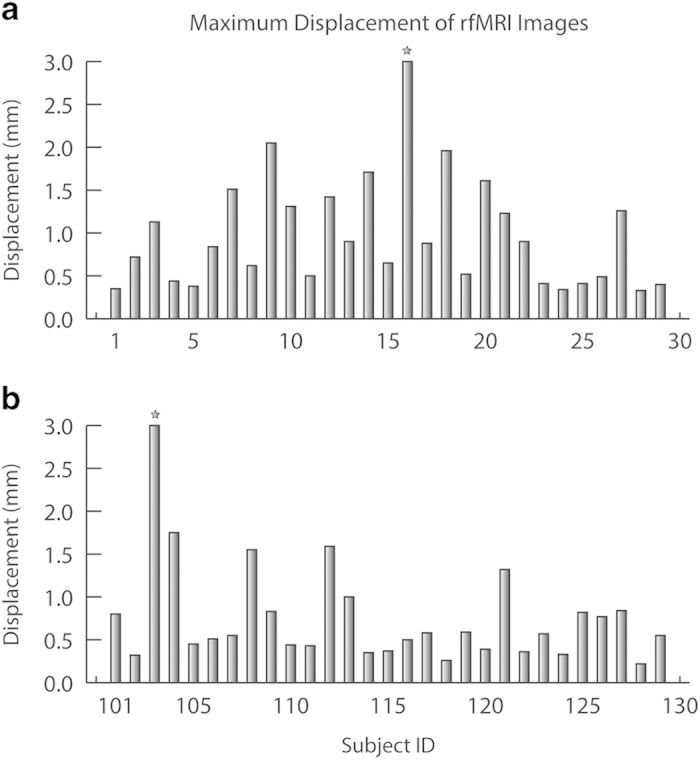
Maximum displacements (mm) of volumes during the fMRI scan. (**a**) the chess group. (**b**) the control group. Note that subject 16 in the chess group had a maximum displacement of 21.19 mm (for visualization purpose, the value was cut off at 3 mm), indicating significant head movements during the fMRI scan. The same happens to subject 103 in the control group, whose maximum displacement was 3.35 mm. The corresponding repository IDs for subject 16 and 103 are 0028212 and 0028299, respectively.

To facilitate the assessment of data quality and image registration performance, we generated snapshots for the items listed in Table 2, and integrated these results in an html webpage, which can be found at each sub-folder of restQA1 (please refer to the data records section for details). In addition, we summarized the CNRs and maximum displacements of rfMRI images for all participants in Figs 2 and 3, respectively. Each figure has two panels, with panel a for the chess group and b for the control group. The averaged CNRs for both groups are 8.81±2.17 and 8.30±0.71 for the chess and control group respectively, and the mean maximum displacements are 0.90±0.53mm and 0.77±0.64mm, respectively. Both measures are typical for 3T EPI fMRI scans. Note that subject 16 (repository ID: 0028212) was excluded in the displacement calculation since this subject has a maximum displacement of 21.19 mm, indicating significant head movements during the fMRI scan. The enormous head movements of this subject introduced strips of hyper intensity in its BOLD images, leading to overestimated *I_max* (please refer to Table 2 for definition) and much higher calculated CNR. No similar issue has been found for the rest of subjects.

### Diffusion weighted imaging (DWI)

There are several QA analysis packages available for DTI data^28–30^. We employed the pipeline (http://www.nitrc.org/projects/masimatlab) provides by Lauzon *et al.* ^28^ in the present study since it covers a comprehensive series of metrics regarding to DTI quality assurance, and is friendly to batch processing. The QA metrics examined in the present study are detailed in Table 3.

To perform the DTI QA analysis, we first aligned the 42 volumes (2 runs, each with 1 b0 image, and 20 DWI images) together using FSL FLIRT^25^. Images of two runs were then averaged, and sent to the pipeline. The pipeline took about 24 h for each subject on our computer, and generated a 4-page pdf report for each subject. These reports are linked in our overview html page. Figue 4 depicts the maximum displacements for each subject during the DTI scan. The mean maximum displacement for the chess group and control group are 2.20±0.79 mm and 2.23±1.09 mm, respectively.

## Usage Notes

Part of this dataset has been successfully used in a few publications^16,18,19^. We encourage researchers to use this dataset in any publication under the requirement of citing this data descriptor. If requested, all scripts used in the present study can be provided. Please also note that we reported QA results for all subjects. Some subjects, e.g., subject 16 (repository ID: 0028212), may have significant head movements. It is the users that will decide whether to exclude a certain subject in their study.

## Additional Information

**How to cite this article:** Li, K. *et al.* A multimodal MRI dataset of professional chess players. *Sci. Data* 2:150044 doi: 10.1038/sdata.2015.44 (2015).

## Supplementary Material



Supplementary File 1

Supplementary File 2

Supplementary File 3

Supplementary File 4

Supplementary File 5

Supplementary File 6

## Figures and Tables

**Figure 4:**
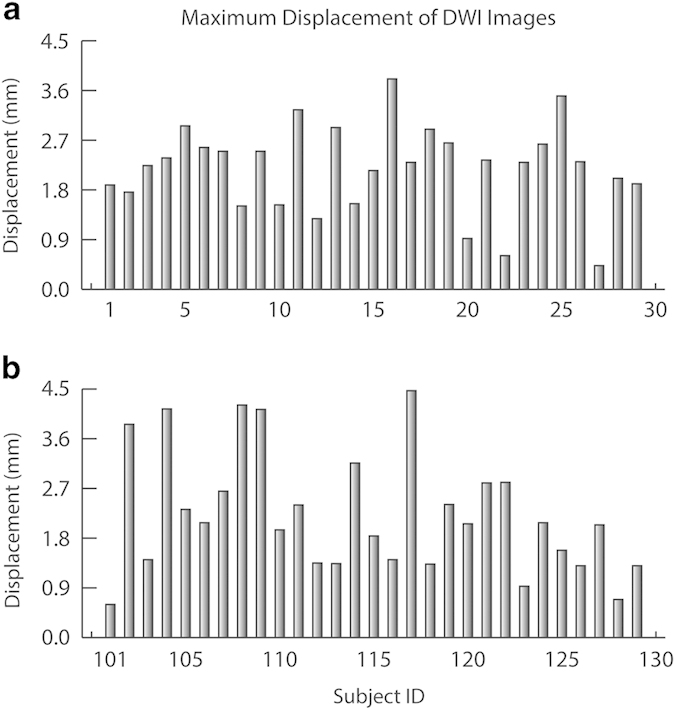
The maximum head displacements of DWI images during the DTI scan using B0 images as references. (**a**) the chess group. (**b**) the control group.

**Table 1:** QA metrics examined in the BXH/XCEDE Tools

**Metrics**	**Notes** *
volmean	The mean intensity of each volume (time point) in the data.
masked_volmean	volmean of the masked and detrended data. Sensitive to spikes.
mean_difference	For a volume vol and a mean volume meanvol, this metric is the mean intensity of (vol—meanvol). Sensitive to slow drifts.
masked_tdiff_volmean	Masked, detrended running difference ('velocity'): this metric subtracts the volmean of a volume from the volmean of its subsequent volume.
cmassx,y and z	Center of mass in x, y, and z directions. Sensitive to head motions.
masked_outlier_percent	The percentage of outlier voxels in each masked volume.
masked_fwhmx,y,z	Full-width half-maximum in x, y and z directions. Used as a measure of data smoothness.
spectrummax	Maximum power for each frequency across all voxels.
per-slice variation	For each slice at each time point in the data, a measure of spikiness at slice granularity that is insensitive to artifacts that affect all slices (e.g., head motion). Higher numbers indicate a spike.
mean	A volume composed of the mean of each voxel across time.
standard deviation	A volume composed of the standard deviation of each voxel across time.
sfnr	Signal-to-Fluctuation Noise Ratio^22^. This is a SNR measure calculated for each brain voxel in the middle slice. It is essentially the average across time divided by standard deviation of detrended signal across time.

*These notes come from the resultant reports using *fmriqa_generate.pl* in BXH/XCEDE.

**Table 2:** rfMRI items examined with our in-house pipeline.

**Categories**	**Items**
**Head motions**	Displacements of each volume in Superior, Left, and Posterior directions.
	Rotations of each volume in degree (roll, pitch, yaw).
**CNR/SNR**	CNR: the amplitude (*I_max*–*I_min*) image divided by the mean standard deviation of non-body background image. For each voxel, *I_max* and *I_min* are the 95% and the 5% percentiles of its intensity histogram across time, respectively.
	Histogram of CNR.
	Temporal SNR or tSNR, similar to Signal-to-Fluctuation Noise Ratio^22^ in Table 1.
**Registration**	highres2standard: the high-resolution sMRI image registered to the MNI152 template space, with the WM/GM edge of the template shown in red.
	func2standard: the rfMRI images registered onto the 3*3*3 mm^3^ MNI152 template.
	func2highres: the rfMRI images registered onto the 1*1*1 mm^3^ T1 sMRI image.
	Segmentation, with GM, WM, and CSF colored and overlaid on the T1 image.
**Other Metrics**	Regional homogeneity^23^. A measure to evaluate local signal homogeneity.
	Amplitude of low-frequency fluctuation or ALFF^24^. Measures the energy of low-frequency fluctuations in rfMRI data.
	Fractional ALFF^26^: Normalized ALFF.

**Table 3:** QA items examined in the DTI pipeline.

**Items**	**Notes**
Translations	Translations of each volume in x, y and z direction.
Rotations	Rotations of each volume in degree (roll, pitch and yaw).
Outliers	Voxels in each DWI scored as outliers by RESTORE^27^.
$\chi_{p}^{2}$	The pixel chi-squared $\chi_{p}^{2}$, a measure of ‘goodness of fit’. Histogram was shown. See ref. 28.
$\chi_{pj-slice}^{2}$	Slice-wise chi-squared $\chi_{p}^{2}$, a measure of fitting per slice. Color map was shown.
Segmentations	Segmentation of DTI images into 25 regions using multiple atlases.
MD	Boxplots of MD per region.
FA	Boxplots of FA, *σ*_*FA*_, and FA bias per region;
Visualizations	MD, FA, *σ*_*FA*_, and FA bias at a mid-Axial slice; mid-axial, mid-sagittal, and mid-coronal views of vector color map with orientation labeling; three mid-axial and three mid-coronal views of the principle vector *e*1 overlaid on vector color map with orientation labeling.
