# Supplementary material for: A multimodal MRI dataset of professional chess players
Source: Sci Data. 2015 Sep 1;2:150044. doi: 10.1038/sdata.2015.44 (PMC4556927; doi:10.1038/sdata.2015.44)
Supplement: Supplementary File 3 [file sdata201544-s4.pdf]

SIEMENS MAGNETOM TrioTim syngo MR B17

\\USER\head\scientific research\Jiangjing\3D-t1_mpr

TA: 7:07 PAT: Off Voxel size: 1.0×1.0×1.0 mm Rel. SNR: 1.00 SIEMENS: tfl

Properties

Prio Recon	Off
Before measurement	
After measurement	
Load to viewer	On
Inline movie	Off
Auto store images	On
Load to stamp segments	Off
Load images to graphic segments	Off
Auto open inline display	Off
Start measurement without further preparation	On
Wait for user to start	Off
Start measurements	single

Routine

Slab group 1	
Slabs	1
Dist. factor	50 %
Position	R2.0 A6.3 F17.9
Orientation	S > C1.5 > T1.2
Phase enc. dir.	A >> P
Rotation	-0.60 deg
Phase oversampling	0 %
Slice oversampling	18.2 %
Slices per slab	176
FoV read	256 mm
FoV phase	100.0 %
Slice thickness	1.00 mm
TR	1900 ms
TE	2.26 ms
Averages	1
Concatenations	1
Filter	Prescan Normalize, Elliptical filter
Coil elements	HEA;HEP;NE2

Contrast

Magn. preparation	Non-sel. IR
TI	900 ms
Flip angle	9 deg
Fat suppr.	None
Water suppr.	None
Averaging mode	Long term
Reconstruction	Magnitude
Measurements	1
Multiple series	Each measurement

Resolution

Base resolution	256
Phase resolution	100 %
Slice resolution	100 %
Phase partial Fourier	7/8
Slice partial Fourier	Off
Interpolation	Off
PAT mode	None
Matrix Coil Mode	Auto (CP)
Image Filter	Off
Distortion Corr.	Off
Unfiltered images	Off
Prescan Normalize	On
Normalize	Off
B1 filter	Off
Raw filter	Off
Elliptical filter	On
Mode	Inplane

Geometry

Multi-slice mode	Single shot
Series	Ascending
Table position	H
Table position	0 mm
Inline Composing	Off

System

Body	Off
NE2	On
HEP	On
HEA	On
SP4	Off
SP2	Off
SP8	Off
SP6	Off
SP3	Off
SP1	Off
SP7	Off
SP5	Off
Positioning mode	REF
MSMA	S - C - T
Sagittal	R >> L
Coronal	A >> P
Transversal	F >> H
Save uncombined	Off
Coil Combine Mode	Adaptive Combine
Auto Coil Select	Default
Shim mode	Tune up
Adjust with body coil	On
Confirm freq. adjustment	Off
Assume Silicone	Off
? Ref. amplitude 1H	0.000 V
Adjustment Tolerance	Auto
Adjust volume	
Position	Isocenter
Orientation	Transversal
Rotation	0.00 deg
R >> L	350 mm
A >> P	263 mm
F >> H	350 mm

Physio

1st Signal/Mode	None
Dark blood	Off
Resp. control	Off

Inline

Subtract	Off
Std-Dev-Sag	Off
Std-Dev-Cor	Off
Std-Dev-Tra	Off
Std-Dev-Time	Off
MIP-Sag	Off
MIP-Cor	Off
MIP-Tra	Off
MIP-Time	Off
Save original images	On

Sequence

Introduction	On
Dimension	3D
Elliptical scanning	Off
Asymmetric echo	Allowed
Bandwidth	200 Hz/Px
Flow comp.	No
Echo spacing	6.8 ms
RF pulse type	Normal
Gradient mode	Normal
Excitation	Non-sel.
RF spoiling	On
