# Supplementary material for: A multimodal MRI dataset of professional chess players
Source: Sci Data. 2015 Sep 1;2:150044. doi: 10.1038/sdata.2015.44 (PMC4556927; doi:10.1038/sdata.2015.44)
Supplement: Supplementary File 4 [file sdata201544-s5.pdf]

SIEMENS MAGNETOM TrioTim syngo MR B17

\\USER\head\function\79-GAD-QIU\rest-mri

TA: 6:54 PAT: Off Voxel size: 3.8×3.8×5.0 mm Rel. SNR: 1.00 SIEMENS: ep2d_pace

Properties

Prio Recon	Off
Before measurement	
After measurement	
Load to viewer	On
Inline movie	Off
Auto store images	On
Load to stamp segments	Off
Load images to graphic segments	Off
Auto open inline display	Off
Start measurement without further preparation	On
Wait for user to start	On
Start measurements	single

Routine

Slice group 1	
Slices	30
Dist. factor	0 %
Position	R4.2 A14.5 H42.8
Orientation	T > C3.1
Phase enc. dir.	A >> P
Rotation	0.00 deg
Phase oversampling	0 %
FoV read	240 mm
FoV phase	100.0 %
Slice thickness	5.0 mm
TR	2000 ms
TE	30 ms
Averages	1
Concatenations	1
Filter	None
Coil elements	HEA;HEP

Contrast

MTC	Off
Flip angle	90 deg
Fat suppr.	Fat sat.
Averaging mode	Long term
Reconstruction	Magnitude
Measurements	205
Delay in TR	0 ms
Multiple series	Off

Resolution

Base resolution	64
Phase resolution	100 %
Phase partial Fourier	Off
Interpolation	Off
PAT mode	None
Matrix Coil Mode	CP
Distortion Corr.	Off
Prescan Normalize	Off
Raw filter	On
Elliptical filter	Off
Hamming	Off

Geometry

Multi-slice mode	Interleaved
Series	Interleaved
Special sat.	None
Table position	H
Table position	0 mm
Inline Composing	Off

System

Body	Off
HEP	On
HEA	On
SP4	Off
SP2	Off
SP8	Off
SP6	Off
SP3	Off
SP1	Off
SP7	Off
SP5	Off
Positioning mode	FIX
MSMA	S - C - T
Sagittal	R >> L
Coronal	P >> A
Transversal	F >> H
Coil Combine Mode	Sum of Squares
Auto Coil Select	Default
Shim mode	Standard
Adjust with body coil	Off
Confirm freq. adjustment	Off
Assume Silicone	Off
? Ref. amplitude 1H	0.000 V
Adjustment Tolerance	Auto
Adjust volume	
Position	R4.2 A14.5 H42.8
Orientation	T > C3.1
Rotation	0.00 deg
R >> L	240 mm
A >> P	240 mm
F >> H	150 mm

Physio

1st Signal/Mode	None

BOLD

GLM Statistics	On
Dynamic t-maps	On
Starting ignore meas	0
Ignore after transition	0
Model transition states	On
Temp. highpass filter	On
Threshold	4.00
Paradigm size	20
Meas[1]	Baseline
Meas[2]	Baseline
Meas[3]	Baseline
Meas[4]	Baseline
Meas[5]	Baseline
Meas[6]	Baseline
Meas[7]	Baseline
Meas[8]	Baseline
Meas[9]	Baseline
Meas[10]	Baseline
Meas[11]	Active
Meas[12]	Active
Meas[13]	Active
Meas[14]	Active
Meas[15]	Active
Meas[16]	Active

Meas[17]	Active
Meas[18]	Active
Meas[19]	Active
Meas[20]	Active
Motion correction	On
Interpolation	3D-K-space
Spatial filter	Off

Sequence

Introduction	Off
Bandwidth	2442 Hz/Px
Free echo spacing	Off
Echo spacing	0.47 ms
EPI factor	64
RF pulse type	Normal
Gradient mode	Fast
