# Supplementary material for: A multimodal MRI dataset of professional chess players
Source: Sci Data. 2015 Sep 1;2:150044. doi: 10.1038/sdata.2015.44 (PMC4556927; doi:10.1038/sdata.2015.44)
Supplement: Supplementary File 5 [file sdata201544-s6.pdf]

SIEMENS MAGNETOM TrioTim syngo MR B17

\\USER\head\function\1--hx\DTI_20_3mm

TA: 5:08 PAT: 2 Voxel size: 1.8×1.8×3.0 mm Rel. SNR: 1.00 SIEMENS: ep2d_diff

Properties

Prio Recon	Off
Before measurement	
After measurement	
Load to viewer	On
Inline movie	Off
Auto store images	On
Load to stamp segments	Off
Load images to graphic segments	Off
Auto open inline display	Off
Start measurement without further preparation	On
Wait for user to start	Off
Start measurements	single

Routine

Slice group 1	
Slices	50
Dist. factor	0 %
Position	R7.0 A23.4 H6.0
Orientation	T > C8.9 > S-0.1
Phase enc. dir.	A >> P
Rotation	-0.80 deg
Phase oversampling	0 %
FoV read	230 mm
FoV phase	100.0 %
Slice thickness	3 mm
TR	6800 ms
TE	93 ms
Averages	2
Concatenations	1
Filter	Raw filter
Coil elements	HEA;HEP

Contrast

MTC	Off
Magn. preparation	None
Fat suppr.	Fat sat.
Averaging mode	Long term
Reconstruction	Magnitude
Delay in TR	0 ms
Multiple series	Off

Resolution

Base resolution	128
Phase resolution	100 %
Phase partial Fourier	6/8
Interpolation	Off
PAT mode	GRAPPA
Accel. factor PE	2
Ref. lines PE	38
Matrix Coil Mode	Auto (Triple)
Reference scan mode	Separate
Distortion Corr.	Off
Prescan Normalize	Off
Raw filter	On
Intensity	Weak
Slope	25
Elliptical filter	Off
Hamming	Off

Geometry

Multi-slice mode	Interleaved
Series	Interleaved
Special sat.	None
Table position	F
Table position	16 mm
Inline Composing	Off

System

Body	Off
HEP	On
HEA	On
SP4	Off
SP2	Off
SP8	Off
SP6	Off
SP3	Off
SP1	Off
SP7	Off
SP5	Off
Positioning mode	REF
MSMA	S - C - T
Sagittal	R >> L
Coronal	A >> P
Transversal	F >> H
Coil Combine Mode	Adaptive Combine
Auto Coil Select	Default
Shim mode	Standard
Adjust with body coil	Off
Confirm freq. adjustment	Off
Assume Silicone	Off
? Ref. amplitude 1H	0.000 V
Adjustment Tolerance	Auto
Adjust volume	
Position	R7.0 A23.4 H6.0
Orientation	T > C8.9 > S-0.1
Rotation	-0.80 deg
R >> L	230 mm
A >> P	230 mm
F >> H	150 mm

Physio

1st Signal/Mode	None
Resp. control	Off

Diff

Diffusion mode	MDDW
Diff. weightings	2
b-value 1	0 s/mm²
b-value 2	1000 s/mm²
Diff. weighted images	On
Trace weighted images	On
Average ADC maps	On
Individual ADC maps	Off
FA maps	On
Mosaic	On
Tensor	On
Noise level	40
Diff. directions	20

Sequence

Introduction	On
Bandwidth	1396 Hz/Px
Free echo spacing	Off

Echo spacing	0.82 ms
EPI factor	128
RF pulse type	Normal
Gradient mode	Fast*
